# Effects and mechanism of dexmedetomidine on neuronal cell injury induced by hypoxia-ischemia

**DOI:** 10.1186/s12871-017-0413-4

**Published:** 2017-08-30

**Authors:** Ya-Jun Liu, Duan-Yu Wang, Yong-Jian Yang, Wei-Fu Lei

**Affiliations:** 1 Department of Anesthesiology, Qilu Hospital of Shandong University, No. 107, Wenhua West Road, Jinan, Shandong 250012 China; 2 Department of Anesthesiology, Jinan Central Hospital Affiliated to Shandong University, Jinan, Shandong 250013 China

**Keywords:** Dexmedetomidine, PC12 cells, Apoptosis, Oxidative stress, Notch/NF-κB pathway

## Abstract

**Background:**

The present study aims to investigate the protective effects of dexmedetomidine (DMED) on hypoxia ischemia injury induced by oxygen and glucose deprivation (OGD) in PC12 and primary neuronal cells.

**Methods:**

PC12 cells exposed to OGD was used to establish ischemia model. The OGD-induced cell injury was evaluated by alterations of cell viability, apoptosis and expressions of apoptosis-associated proteins. Oxidative stress and expressions of neurotrophic factors after OGD and DMED treatments were also explored. The activation of possible involved signaling pathways were studied after OGD and DMED treatments, along with the addition of inhibitors of these pathways. Finally, the effects of DMED on primary neuronal cells were verified according to the alterations of inflammatory cytokines release and oxidative stress.

**Results:**

DMED obviously increased cell viability and reduced cell apoptosis as well as ratio of Bax/Bcl-2 in OGD-treated PC12 cells. Then, the OGD-induced changes of LDH, MDA, SOD and GSH-Px as well as decreases of neurotrophic factors were all ameliorated by DMED treatment. Key kinases in Notch/NF-κB signaling pathway were up-regulated by OGD, whereas the up-regulations were decreased by DMED. In addition, inhibitor of Notch or NF-κB could augment the effects of DMED on OGD-induced cell injury. Finally, the protective effects of DMED were verified in primary neuronal cells.

**Conclusion:**

DMED had protective effect on OGD-induced PC12 cell injury, depending on its anti-apoptotic, anti-oxidative activity and the inhibition of Notch/NF-κB activation. Our findings suggested that DMED could be used as a potential therapeutic drug for cerebral ischemia.

## Background

As a common cerebrovascular disease, ischemic stroke is the most common cause of death and adult disability all over the world [1]. Ischemic stroke is characterized by the disturbance of the blood supply to the brain [2]. A blockage of blood flow in an artery, caused by a thrombus or embolus, leads to a sudden reduction of blood flow, resulting in failure of delivering glucose, oxygen, and nutrient, which are important, to the brain [3]. Although thrombolysis becomes an effective agent for the treatment of ischemic stroke, the narrow therapeutic time window makes this agent useless for large proportion of patients [4]. Thus, novel therapeutic methods are urgently needed for the treatment of ischemic stroke.

Neuronal injury induced by cerebral ischemia is a complex process with various mechanisms, including oxidative stress, apoptosis, activation of kinases and changes in gene expression [5, 6]. Oxidative stress plays a critical role in neuronal injury and has close connections with the pathogenesis of various central nervous system diseases such as cerebral ischemia, Parkinson’s disease and Alzheimer’s disease. Accumulating evidence has reported that oxidative stress could be a therapeutic target of cerebral ischemia [7]. Oxygen-glucose deprivation (OGD) is an in vitro cell model that simulates the in vivo ischemia for exploring the mechanisms of neuronal damage induced by ischemia and for the discovery of the potential protective agents for the treatment of ischemia [8, 9].

PC12 cell line, derived from rat pheochromocytoma, has been widely used for cell signaling studies, such as cerebral ischemia and neuronal protection studies [10, 11]. PC12 cells possess the properties of sympathetic neurons with the impact of nerve growth factor [12]. Many studies focused on neurotoxicity [13], amyloid β-induced cell apoptosis [14] and Alzheimer’s disease [15] have been performed by using PC12 cells.

Dexmedetomidine (DMED) is a new type of clinical anesthetic agent, which is used for calmness of tracheal intubation and mechanical ventilation in surgical patients under general anesthesia. Some studies have discovered that DMED has neuroprotective effects with the preconditioning and attenuation of ischemia injury [16, 17]. Another study has reported that DMED could decrease cerebral blood flow [18]. However, the possible involved mechanisms remain unclear. This study was aimed to uncover the neuronal protective mechanisms of DMED in PC12 cells or primary hippocampal neuronal cells with OGD.

## Methods

### Cell culture of PC12 and primary hippocampal neuronal cells

PC12 cells (American Type Culture Collection, Manassas, VA, USA) were cultured in DMEM containing L-glutamine, 10% fetal bovine serum, 100 U/mL penicillin, 5% horse serum, and 100 μg/mL streptomycin (all from Invitrogen). Cell culture of PC12 cells was performed in humidified atmosphere of 5% CO_2_ and 95% air at 37 °C. Primary hippocampal neuronal cells were isolated from neonatal Wistar rats (2–3 d old, male) following the methods described previously [19]. In brief, bilateral hippocampi were isolated carefully, followed by mechanical fragmentation and digestion with 0.25% trypsin at 37 °C for 20 min. After centrifugation at 200×*g* for 5 min, the isolated cells were collected and re-suspended in Dulbecco’s modified Eagle medium and Ham’s F-12 medium (DMEM/F12; Invitrogen, Burlington, ON, Canada) supplemented with 20% fetal bovine serum (Invitrogen). Then, the re-suspension containing neurons at a density of 1.0 × 10^6^ cells/mL was planted into a cell culture flask, which was pre-coated with 0.1 mg/mL polyL-lysine (Sigma-Aldrich, St. Louis, MO, USA), and these neurons were cultured at 37 °C in humidified air with 5% CO_2_. The culture medium was exchanged with neurobasal medium supplemented with 2% B27, 10 mM glutamine and 1 mM sodium pyruvate. All the procedures were approved by the Ethical Committee of Qilu Hospital of Shandong University.

### Cell treatments

To establish OGD-induced hypoxic-ischemic model, the cells were cultured in the DMEM culture medium without glucose, and then incubated in an oxygen-free chamber with 95% N_2_ and 5% CO_2_ for 4 h at 37 °C. There were three different groups in the experiments: (1) control, (2) OGD exposure and (3) OGD exposure plus treatment with DMED. The control group was always maintained in normal DMEM and put in the incubator under normoxic conditions. To verify the possible involved signaling pathway, DAPT (Notch1 inhibitor, 10 μM; Sigma-Aldrich) and SN50 (NF-κB inhibitor, 25 μg/mL; Sigma-Aldrich) were respectively added into PC12 cells, which were treated with OGD and DMED, followed by measurement of cell viability, apoptosis and oxidative stress.

### Cell viability

Cell viability was analyzed by 3-(4,5-dimethylthiazol-2-yl)-2,5-diphenyltetrazolium bromide (MTT) assay [20]. After a period of incubation, PC12 cells (100 μL), diluted to 1 × 10^5^ cells/mL, were seeded into each well of a 96-well micro-plate. After treatments, MTT solution was added to the cells with a final concentration of 1 mg/mL and the mixture was allowed to incubate at 37 °C for 4 h. Then the supernatant was removed by centrifuging, and the pellets were dissolved in dimethyl sulfoxide (DMSO). The absorbance was measured by spectrophotometry at 570 nm using an Elx-800uv reader (Bio-Tek Instruments, Winooski, VT, USA).

### Cell apoptosis

Cell apoptosis was assessed by Terminal deoxynucleotidyl transferase (TdT)-mediated dUTP nick end labeling (TUNEL) staining using an in situ cell death detection kit (KeyGEN, Nanjing, China). After treatment, PC12 cells were fixed by 4% paraformaldehyde at 4 °C for 30 min, followed by rinsing with phosphate-buffered saline (PBS). Then, PC12 cells were stained according to the manufacture’s information. The number of cells was counted under microscopic observation. Cell apoptosis was expressed as the ratio of the number of TUNEL-positive cells to the total number of cells, which were counted in five randomly chosen fields.

### Quantitative reverse transcription real-time quantitative PCR analysis (qRT-PCR)

The total RNA of PC12 cells after treatment was isolated using Trizol reagent (Invitrogen) according to the manufacturer’s instructions. RNA quality and quantity was measured using a nanodrop spectrophotometer (ND-1000, Nanodrop Technologies, Wilmington, DE, USA). RNA was reversely transcribed into cDNA using a Multiscribe RT kit (Applied Biosystems, Foster City, CA, USA) in accordance with the recommendation of supplier. Quantification of mRNA expression was performed with Fast SYBR Green (Applied Biosystems) following the supplier’s protocol. Primers were designed and synthesized by Sangon Biotech (Shanghai, China). On the basis of the method described previously [21], relative expression of genes were calculated, normalizing to GAPDH.

### Western blot analysis

PC12 cells were lysed with lysis buffer and incubated on ice for 30 min. Then the lysate was centrifuged at 10000×*g* at 4 °C for 20 min, and the supernatant were quantified with BCA™ Protein Assay Kit (Pierce, Rockford, IL, USA). Proteins (30 μg/lane) of PC12 cells were subjected to SDS-PAGE and were then transferred to PVDF membranes. The blots were incubated with primary antibodies against B cell lymphoma-2 (Bcl-2, ab194583), Bcl-2-associated X protein (Bax, ab182733), brain-derived neurotrophic factor (BDNF, ab205067), nerve growth factor (NGF, ab52918), Nestin (ab6142), nuclear factor κB (NF-κB, ab28856), inhibitor of NF-κB α (IκBα, ab32518), GAPDH (ab181603) (all from Abcam, Cambridge, UK), Notch intracellular domain (NICD; 3608, Cell Signaling Technology, Beverly, MA, USA) or Notch1 (sc-32,756; Santa Cruz, Santa Cruz, CA, USA) overnight at 4 °C, and then incubated with secondary antibodies conjugated to horseradish peroxidase at room temperature for 1 h. The bound antibody proteins were visualized by an enhanced chemiluminescence detection system (GE Healthcare Biosciences, Piscataway, NJ, USA). The signals were captured and the intensity of the bands was quantified using Image Lab™ software (Bio-Rad, Shanghai, China).

### Evaluation of oxidative injury

Oxidative stress-induced cell injury was evaluated according to the alterations of lactate dehydrogenase (LDH), malordiaolehyde (MDA), superoxide dismutase (SOD) and glutathione peroxidase (GSH-Px). The release of LDH into the medium was measured by LDH assay kit (Bio Vision Inc., Milpitas, CA, USA) at 440 nm. The percentage of LDH leakage was expressed as culture medium OD values / (culture medium OD values + cells homogenate OD values) × 100%. For assay of free radicals, the cells were washed with ice-cold PBS. The content of MDA was determined with a Cell MDA assay kit (Jiancheng Bioengineering Institute, Nanjing, China) according to the manufacture’s protocol. The absorbance of each sample was measured at 530 nm. SOD activity was measured by the xanthine-xanthine oxidase method. The supernatant was added to xanthine-xanthine oxidase reagent and the mixture was incubated for 40 min at 37 °C. SDS was added to stop the reaction, followed by measurement of absorbance at 550 nm. GSH-Px activity was measured with Glutathione Peroxidase Assay Kit (Jiang Lai biology, Shanghai, China) following the protocol of supplier.

### Detection of tumor necrosis factor-α (TNF-α), interleukin (IL)-6, NADPH oxidase 2 (Nox2) and catalase (CAT) in the hippocampus

Primary neuronal cells were divided into three groups, namely control, OGD and OGD + DMED groups. After treatments, culture media was collected for measurements of TNF-α and IL-6 using commercially available enzyme-linked immunosorbent assay (ELISA) kits (R&D Systems, Minneapolis, MN, USA). The concentration of Nox2 in primary neuronal cells was determined by using an ELISA kit purchased from MyBioSource (San Diego, CA, USA). CAT activity was assessed by using chemical assay kits (Jiancheng Bioengineering Institute).

### Statistical analysis

Results are presented as mean ± SD of at least three independent experiments. Statistical analysis was performed with the software package SPSS 10.0 (SPSS Inc., Chicago, IL, USA). The significance of differences was determined by two-way analysis of variance (ANOVA) or unpaired two-tailed t-test. The *p* values less than 0.05 were considered to be statistically significant.

## Results

### Effects of DMED on cell viability and apoptosis in PC12 cells under OGD

**Fig. 1:**
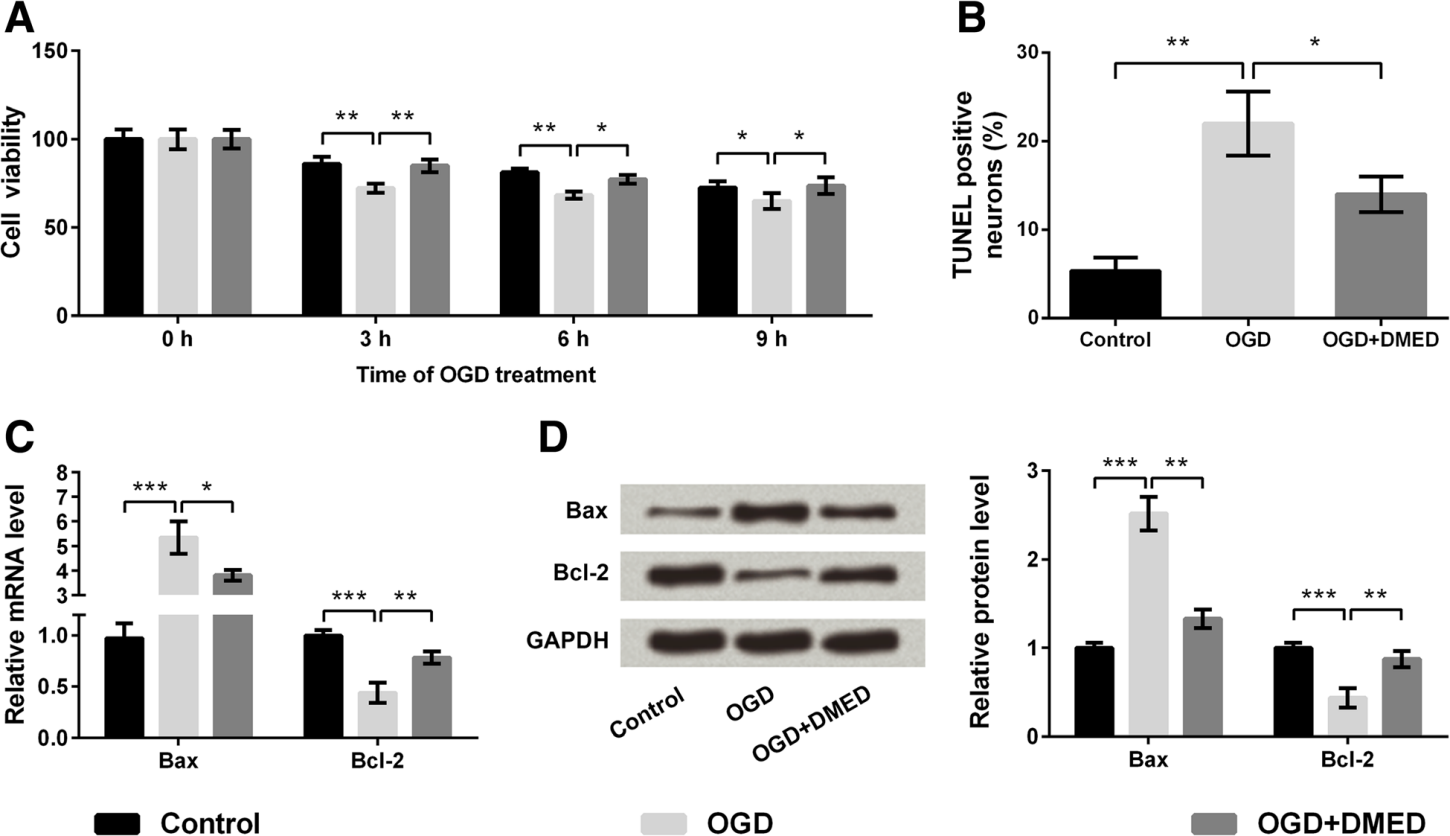
Effects of dexmedetomidine (DMED) on cell viability and apoptosis of PC12 cells treated with oxygen-glucose deprivation (OGD). **a** Cell viability. **b** Cell apoptosis. **c** mRNA expressions of Bax and Bcl-2. **d** Protein expression of Bax and Bcl-2. Bcl-2, B cell lymphoma-2; Bax, Bcl-2-associated X protein. Data are presented as the mean ± SD. ^*^ *p* values <0.05; ^**^ *p* values <0.01; ^***^ *p* values <0.001

The results in Fig. 1a showed that OGD treatment time-dependently decreased the viability of PC12 cells. Compared with control group, cell viability was significantly decreased at 3 h (*p* < 0.01), 6 h (*p* < 0.01) and 9 h (*p* < 0.05) after OGD treatment. Interestingly, the OGD-induced reduction of cell viability was significantly alleviated by DMED at 3 h (*p* < 0.01), 6 h (*p* < 0.05) and 9 h (*p* < 0.05) after OGD. Apoptosis is considered to play a critical role in brain ischemia injury [22]. TUNEL assay was used to analyze apoptosis in PC12 cells. As shown in Fig. 1b, OGD significantly promoted cell apoptosis compared to control group (*p* < 0.01), while DMED obviously alleviated the OGD-induced increase of TUNEL-positive cells in PC12 cells (*p* < 0.05). The qRT-PCR and western blot analysis were respectively used to detect the mRNA and protein levels of Bax and Bcl-2. The results showed that the expression of Bax was significantly increased and the expression of Bcl-2 was significantly decreased after the treatment of OGD at both mRNA and protein levels (*p* < 0.001). However the administration of DMED obviously alleviated the effect of OGD on PC12 cells compared with OGD group (*p* < 0.05 or *p* < 0.01; Fig. 1c-d).

### Effects of DMED on oxidative stress in PC12 cells under OGD

**Fig. 2:**
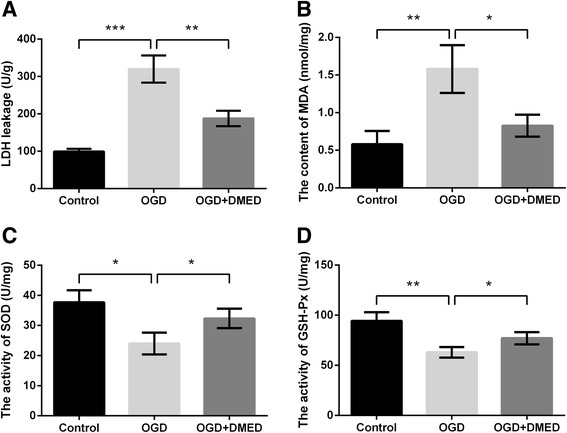
Effects of dexmedetomidine (DMED) on oxidative stress in PC12 cells treated with oxygen-glucose deprivation (OGD). **a** Lactate dehydrogenase (LDH) leakage. **b** Content of malordiaolehyde (MDA). **c** Activity of superoxide dismutase (SOD). **d** Activity of glutathione peroxidase (GSH-Px). Data are presented as the mean ± SD. ^*^ *p* values <0.05; ^**^ *p* values <0.01; ^***^ *p* values <0.001

Oxidative stress induced by OGD can lead to the death of PC12 cells [23], thus the related indicators of oxidative stress, including LDH, MDA, SOD and GSH-Px were all evaluated. As shown in Fig. 2a-b, OGD treatment significantly increased LDH leakage into the medium and the content of MDA compared with control group (*p* < 0.01 or *p* < 0.001), while DMED markedly attenuated the increases compared with OGD group (*p* < 0.05 or *p* < 0.01). Conversely, the activity of SOD and GSH-Px were both reduced by OGD compared with control group (*p* < 0.05 or *p* < 0.01), whereas these reductions were ameliorated by DMED compared with OGD group (*p* < 0.05, Fig. 2c-d).

### DMED promoted the repair of neurons after ischemia anoxic injury

**Fig. 3:**
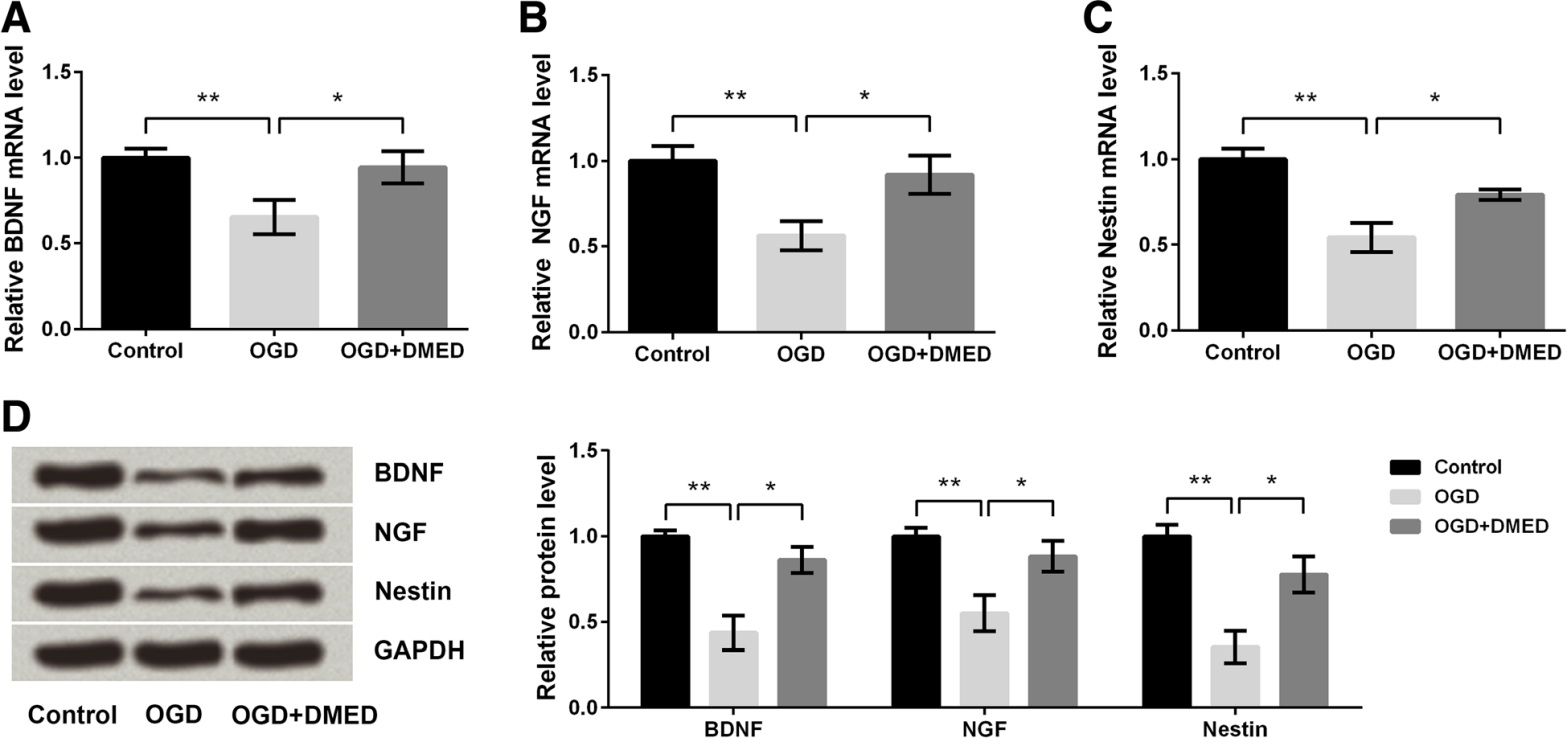
Effects of dexmedetomidine (DMED) on the repair of neurons after the treatment with oxygen-glucose deprivation (OGD). **a** The mRNA level of brain-derived neurotrophic factor (BDNF). **b** The mRNA level of nerve growth factor (NGF). **c** The mRNA level of Nestin. **d** Protein levels of BDNF, NGF and Nestin. Data are presented as the mean ± SD. ^*^ *p* values <0.05; ^**^ *p* values <0.01

The expressions of BDNF, NGF, and Nestin were analyzed by qRT-PCR and western blot analysis. The results showed that OGD treatment significantly decreased the mRNA levels of BDNF, NGF and Nestin in PC12 cells compared with control group (*p* < 0.01). However, DMED alleviated the OGD-induced decreases of BDNF, NGF and Nestin levels compared with OGD group (*p* < 0.05), indicating that DMED promoted the repair of neuronal impairment induced by OGD (Fig. 3a-c). Results in Fig. 3d also proved the protective effect of DMED on neuronal impairment.

### Notch/NF-κB was involved in the protection of DMED on OGD-induced nerve injury

**Fig. 4:**
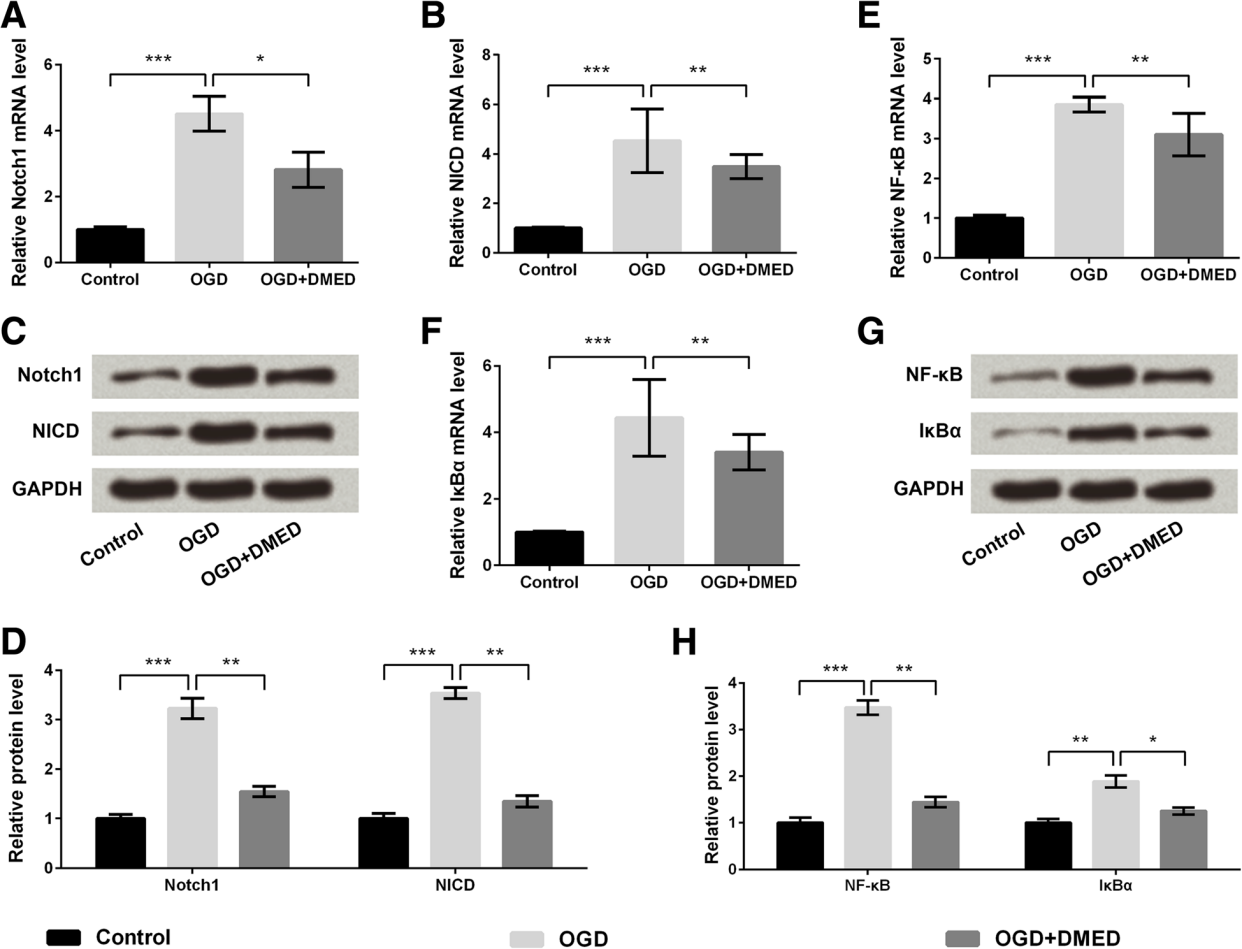
Notch/nuclear factor κB (NF-κB) pathway was involved in the protective effect of dexmedetomidine (DMED) on oxygen-glucose deprivation (OGD)-induced neurons. **a** The mRNA level of Notch1. **b** The mRNA level of Notch intracellular domain (NICD). **c** The protein expressions of Notch1 and NICD. **d** The relative protein levels of Notch1 and NICD. **e** The mRNA level of NF-κB. **f** The mRNA level of inhibitor of NF-κB α (IκBα). **g** The protein expressions of NF-κB and IκBα. **h** The relative protein levels of NF-κB and IκBα. Data are presented as the mean ± SD. ^*^ *p* values <0.05; ^**^ *p* values <0.01; ^***^ *p* values <0.001

The expressions of key kinases involved in Notch and NF-κB signaling pathways were analyzed by qRT-PCR and western blot analysis. The results showed that OGD treatment significantly increased the mRNA and protein levels of Notch1 and NICD compared with control group (*p* < 0.001), but the OGD-induced up-regulations of Notch1 and NICD were alleviated by DMED (*p* < 0.05 or *p* < 0.01), indicating that DMED inhibited the OGD-induced activation of Notch pathway (Fig. 4a-d). Similarly, the marked up-regulations of NF-κB and IκBα at mRNA and protein levels after treatment of OGD (*p* < 0.0﻿1﻿ or﻿﻿ *p* < 0.001) were both significantly alleviated by DMED treatment (*p* < 0.05 or *p* < 0.01), indicating that DMED inhibited the OGD-induced activation of NF-κB pathway (Fig. 4e-h).

### Inhibition of Notch or NF-κB pathway augmented the protective effect of DMED on OGD-induced cell injury

**Fig. 5:**
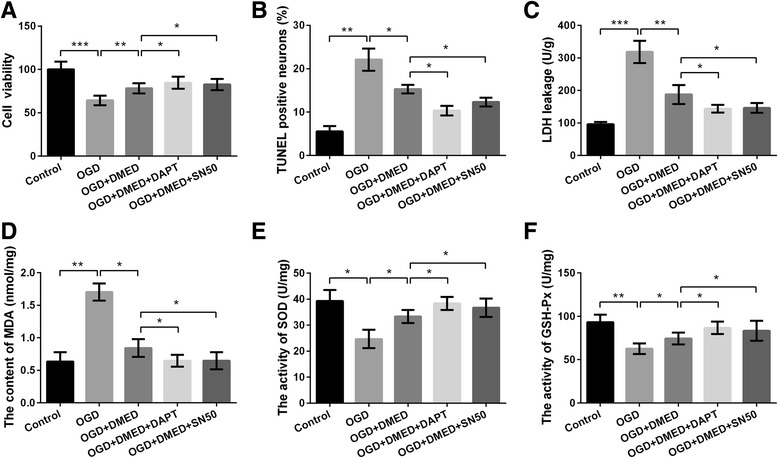
Inhibitor of Notch or NF-κB augmented the protective effects of dexmedetomidine (DMED) on oxygen-glucose deprivation (OGD)-induced PC12 cells. **a** Cell viability. **b** Cell apoptosis. **c** Lactate dehydrogenase (LDH) leakage. **d** Content of malordiaolehyde (MDA). **e** Activity of superoxide dismutase (SOD). **f** Activity of glutathione peroxidase (GSH-Px). Data are presented as the mean ± SD. ^*^ *p* values <0.05; ^**^ *p* values <0.01; ^***^ *p* values <0.001

The effects of DMED on OGD-induced cell injury were assessed again with or without the presence of Notch inhibitor (DAPT) or NF-κB inhibitor (SN50). Results in Fig. 5a and e-f showed cell viability at 6 h after OGD as well as activities of SOD and GSH-Px was enhanced by treatment of DAPT or SN50 compared with OGD + DMED group (*p* < 0.05). Cell apoptosis as well as levels of LDH and MDA was markedly reduced by treatment of DAPT or SN50 compared with OGD + DMED group (*p* < 0.05, Fig. 5b-d). Results indicated that inhibition of Notch or NF-κB pathway could enhanced the protective effects of DMED on OGD-induced cell injury in PC12 cells.

### OGD-induced cell injury in primary neuronal cells was alleviated by DMED

**Fig. 6:**
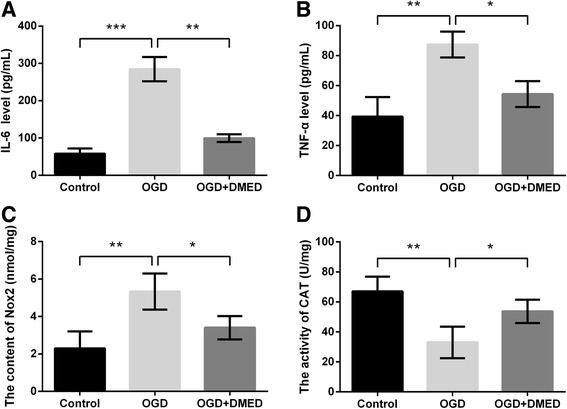
Effects of dexmedetomidine (DMED) on primary neuronal cells treated with oxygen-glucose deprivation (OGD). **a** The level of interleukin (IL)-6. **b** The level of tumor necrosis factor-α (TNF-α). **c** The level of NADPH oxidase 2 (Nox2). **d** The level of catalase (CAT). Data are presented as the mean ± SD. ^*^ *p* values <0.05; ^**^ *p* values <0.01; ^***^ *p* values <0.001

The protective effects of DMED on OGD-induced cell injury were verified in primary neuronal cells. The cell injury was evaluated according to the alterations of IL-6, TNF-α, Nox2 and CAT. Results in Fig. 6a-c showed levels of IL-6, TNF-α and Nox2 were elevated by OGD (*p* < 0.01 or *p* < 0.001), whereas the increases were alleviated by DMED (*p* < 0.05 or *p* < 0.01). Conversely, OGD decreased activity of CAT (*p* < 0.01) but the decrease was alleviated by DMED (*p* < 0.05).

## Discussion

DMED is a highly selective, short acting central α_2_-adrenergic receptor agonist, which binds to transmembrane G protein-binding receptors in brain and spinal cord tissues [24, 25]. It has been reported that DMED has neuroprotective effects [26]. Therefore in the present study, we explored the neuroprotective effects of DMED on OGD-induced ischemic injury in PC12 cells. The degree of cell injury induced by OGD was measured by MTT assay and LDH release in this study. MTT is a substrate for intracellular and cellular membrane oxidoreductases [27], and the number of its reductive form has close relationship with the amount of living cells. Some studies have found that the degree of LDH leakage has correlation with cell membrane damage [27, 28]. In this study, the decreased cell viability and elevated levels of LDH were discovered in PC12 cells treated with OGD, which was in accordance with the results in a previous study [29]. However, the administration of DMED significantly alleviated the reduction of cell viability and inhibited the release of LDH, indicating that DMED improved OGD-induced injury in PC12 cells.

Apoptosis is a form of cell death that is mediated by many factors, including oxidative stress, excitotoxicity and so on [30, 31]. Bcl-2 and Bax, the members of the Bcl-2 family of proteins, can mediate apoptosis and attenuate caspase-3 activation [32]. To further understand the mechanism of apoptosis, the expression levels of Bcl-2 and Bax were analyzed in our study. The results showed that after OGD-treatment, the mRNA and protein expression of Bax was significantly increased and that of Bcl-2 was decreased. However, the treatment of DMED nearly reserved the levels of Bcl-2 and Bax to normal, providing an explanation for the effect of DMED on cell apoptosis after OGD.

Available studies have discovered that oxidative stress plays a critical role in the pathogenesis of many neurological diseases such as Alzheimer’s disease and stroke [33]. Lipid peroxidation and oxygen free radical play an important role in the development of injuries in the central nervous system. SOD is usually considered to be the important line of defense against cell injury caused by cytotoxic reactive oxygen species [34]. The results in this study showed that OGD-treatment led to an obvious increase of MDA and a decrease of SOD and GSH-Px activity. Our results were in accordance with a previous research, which discovered decreased SOD and GSH-Px activity in the ischemic rats [35]. However, the treatment of DMED alleviated the enhancement of MDA and increased the activities of SOD and GSH-Px.

Neurotrophic factors, including BDNF, NGF and Nestin, are essential for the development and survival of neurons, as well as the maintenance and plasticity of brain [36]. BDNF, NGF or combination of these two factors were reported to induce neuronal differentiation of neural stem cells [37]. In our study, expressions of these three factors were all down-regulated by OGD whereas the down-regulations were all ameliorated by DMED treatment, indicating the neuroprotection of DMED.

Notch proteins are a family of single-pass transmembrane heterodimeric receptors, which participate in cell proliferation or differentiation in many tissues [38]. Notch signaling pathway is one of the most conserved pathways with plenty of functions [39], which can regulate the survival and apoptosis of neural stem cells during the development of the brain. The expression and activation of Notch signaling have been reported in different diseases. Increasing evidence discovered that the disorders of Notch signaling were the main cause for the activation of many oncogenic pathways, such as NF-κB [40, 41]. The results in the present study showed that OGD treatment significantly increased the levels of Notch1 and NICD, indicating that OGD activated the Notch pathway, while DMED inhibited the activation of Notch pathway. The similar results were also found in NF-κB pathway, indicating that Notch/NF-κB pathway was involved in the protection of DMED on OGD-induced nerve injury. Further studies performed with Notch or NF-κB inhibitor proved the involvements of the Notch/NF-κB pathway in the neuroprotection of DMED in OGD-induced neuronal injury.

TNF-α and IL-6 are two inflammatory factors that could be induced by hypoxia [42]. Nox, expressed throughout the central nervous system, are a major source of brain ROS [43]. CAT is part of endogenous anti-oxidant system and its up-regulation indicates enhancement of anti-oxidant system [44]. In our study, the protective effects of DMED were also proved in the primary neuronal cells. The OGD-induced increases of IL-6, TNF-α and Nox2 level as well as decrease of CAT activity were all ameliorated by DMED treatment, consolidating the neuroprotection of DMED in OGD-induced cell injury.

## Conclusion

In summary, DMED had protective effect on OGD-induced cell injury of PC12 and primary neuronal cells. Such protection of cells against ischemia might depend on its anti-apoptotic and anti-oxidative activity and the inhibition of Notch/NF-κB activation. Our findings suggested that DMED could be used as a potential therapeutic for cerebral ischemia.

## Abbreviations

DMED: Dexmedetomidine
DMSO: Dimethyl sulfoxide
GSH-Px: Glutathione peroxidase
LDH: Lactate dehydrogenase
MDA: Malordiaolehyde
OGD: Oxygen and glucose deprivation
SOD: Superoxide dismutase
TdT: Terminal deoxynucleotidyl transferase
